# Presence of galactosylated core fucose on N-glycans in the planaria *Dugesia japonica*

**DOI:** 10.1002/jms.1925

**Published:** 2011-06

**Authors:** Katharina Paschinger, Ebrahim Razzazi-Fazeli, Kiyoshi Furukawa, Iain BH Wilson

**Affiliations:** a Department für Chemie, Universität für BodenkulturMuthgasse 18, A-1190 Wien, Austria; b Vetomics Core Facility for Research, Veterinärmedizinische UniversitätA-1210 Wien, Austria; c Department of Bioengineering, Nagaoka University of TechnologyNagaoka 940-2188, Japan

**Keywords:** Galβ1,4Fuc epitope, planaria, N-glycan, methylhexose, mass spectrometry

## Abstract

Planarial species are of especial interest to biologists due to the phenomenon of pluripotency and, in comparison to other developmental processes, it can be hypothesised that glycan–lectin interactions may play a role. In order to examine the N-glycans of one of these organisms, *Dugesia japonica*, peptide:N-glycosidase A was employed and the released glycans were subject to pyridylamination, HPLC and mass spectrometric analysis. A range of oligomannosidic glycans was observed with a trimethylated Man_5_GlcNAc_2_ structure being the dominant species. Three glycans were also observed to contain deoxyhexose; in particular, a glycan with the composition Hex_4_HexNAc_2_Fuc_1_Me_2_ was revealed by exoglycosidase digestion, in combination with MS/MS, to contain a galactosylated core α1,6-fucose residue, whereas this core modification was found to be capped with a methylhexose residue in the case of a Hex_5_HexNAc_2_Fuc_1_Me_3_ structure. This is the first report of these types of structures in a platyhelminth and indicates that the ‘GalFuc’ modification of N-glycans is not just restricted to molluscs and nematodes. Copyright © 2011 John Wiley & Sons, Ltd.

## INTRODUCTION

Glycans cover the surfaces of all cells and, therefore, it is expected that many cell–cell interactions, whether between the cells of an individual organism or between symbionts or pathogens and their hosts, are glycan dependent.[1] As some of these glycan-based cell–cell interactions are of developmental relevance, it is of interest to identify paradigmatic examples of simple and/or tractable developmental systems, examples being those of slime moulds, nematodes or insects.[2] The planaria represent another developmental model, whose properties are remarkable in the context of cell programming, as by means of pluripotent neoblasts, a large degree of regeneration of a wounded animal is possible.[3,4] However, despite the general biological knowledge about planaria and the identification of some differentially expressed potential C-type lectins in one species, *Girardia tigrina*,[5] and the use of lectins as histochemical tools in another, *Schmidtea mediterranea*,[6] very little is known about the glycogenomic potential of these organisms. Therefore, we examined the N-glycans of one of them, *Dugesia japonica*; such data are a pre-requisite before embarking on studies examining whether the glycome has a role in the special regenerative properties of planarian species. By the use of MS/MS and exoglycosidase digestion, we extend data recently published on the N-glycans of this organism.[7]

## EXPERIMENTAL PROCEDURES

### Sample preparation

The planarians (*D. japonica*) were harvested from local fresh water streams in Japan and cultured in water at 15 °C in the dark. They were occasionally fed with chicken liver. Before harvesting, they were starved for a week, washed with phosphate-buffered saline (pH 7.4) several times and subjected to homogenisation (dissected and then sonicated) in 10 mm phosphate buffer, pH 7.0. The pellet was isolated by centrifugation (3000 *g*, 15 min, 4 °C) and suspended in acetone/water (1:1, v/v) several times. The final pellet suspended in water was subjected to lyophilisation. Glycopeptides were prepared from the lyophilised material (6 mg) using pepsin, basically as previously described[8]; after initial ion-exchange (Dowex 50 W × 8, Sigma–Aldrich) and gel filtration (Sephadex G25, GE Healthcare) chromatography, the sample was dissolved in 50 µl of 5% (v/v) ammonia. In order to avoid later fluorescent labelling of residual non-N-glycan free oligosaccharides in the mixture (see also Section on Results), the sample was pre-reduced with 50 µl of 1% (w/v) sodium borohydride at room temperature for 2 h prior to the addition of 2 µl of acetic acid and subsequent lyophilisation. The N-linked glycans were released using peptide:N-glycosidase A (Roche). Thereafter, the sample was subject to Dowex 50 W × 8 cation exchange chromatography; the unbound fraction was pyridylaminated at pH 7[9] and excess reagent was removed by gel filtration (Sephadex G15, GE Healthcare).

### HPLC and MALDI-TOF MS analyses

The labelled N-glycans were analysed by reversed-phase and normal-phase HPLC using, respectively, an MZ Analytik ODS Hypersil and a Takara Palpak type N column[10] on a Shimadzu HPLC system equipped with a fluorescence detector (RF 10 AXL). In the case of RP-HPLC, a linear gradient of 0.3%/min methanol in 0.1 m ammonium acetate, pH 4, was applied. For NP-HPLC, buffer A was a 25:75 mixture of 3% acetic acid adjusted with triethylamine and acetonitrile, whereas buffer B was a 50:50 mixture. The gradient of buffer B was applied as follows: 0–5 min, 10% B; 5–45 min, 10–100% B; 45–50 min, 100% B; followed by a return to the starting conditions. The columns were calibrated with an pyridylaminated oligoglucose standard as well as with N-glycans prepared from *Drosophila melanogaster* S2 cells.[11] Glycans were detected by fluorescence (excitation, 310 or 320 nm; emission, 380 or 400 nm). In the case of the RP-HPLC, each fraction was lyophilised and dissolved in 10 µl; 1 µl thereof was dried on a steel sample plate under vacuum before applying either 2,5-dihydroxybenzoic acid or 6-aza-2-thiothymine as matrix, which was again dried under vacuum. The samples were then analysed in positive mode by MALDI-TOF MS using a Bruker Ultraflex I equipped with a nitrogen laser (337 nm; laser frequency of 50 Hz and pulse length of 200 ns); typically 400–1000 shots were summed. Selected species were further examined by MS/MS (post-source decay).

### Exoglycosidase digestion

An aliquot of fraction **X** (1 µl) was mixed with 0.5 µl of 0.1 m ammonium acetate, pH 5, buffer in a PCR tube together with either bovine α-fucosidase (10 mU), *Aspergillus oryzae* β1,4-galactosidase (70 mU) or a combination of the two enzymes and incubated overnight at 37 °C, prior to MALDI-TOF MS and MS/MS analysis with 6-aza-2-thiothymine as the matrix.

## RESULTS

### Overall N-glycomic analysis of *Dugesia japonica*

**Figure 1:**
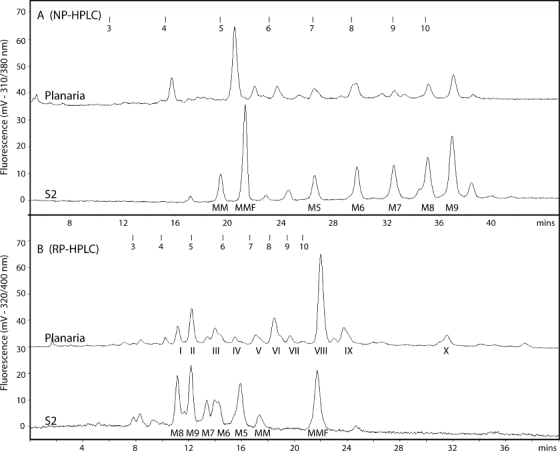
Chromatographic analysis of pyridylaminated *Dugesia* N-glycans. Normal phase (NP) and reverse phase (RP) HPLC of planaria glycans were performed in comparison to an isomalto-oligosaccharide series (3–10 g.u.) and a preparation of N-glycans from *Drosophila* S2 cells. The ten collected RP-HPLC fractions are indicated in roman numerals, while the major fractions of S2 cells are annotated according to the glycans detected by MALDI-TOF MS: MM/MMF (Man_3_GlcNAc_2_Fuc_0–1_) and M5/M6/M7/M8/M9 (Man_5–9_GlcNAc_2_).

The basic procedures for the analysis of the N-glycans of the planaria *D. japonica* were performed as with other organisms; however, as initial studies indicated that a polyhexose series of unknown origin was present, a subsequent preparation was subject to reduction prior to the release of the N-glycans, so that only the released glycans and not the polyhexose compounds could be later labelled by the reductive pyridylamination method. Subsequent NP-HPLC analysis, using an isomalto-oligosaccharide series and a sample of N-glycans of insect S2 cells[11] as standards, indicated that *D. japonica* expressed a range of glycans co-eluting, in part, with an oligomannosidic series (Fig. 1(A)). However, the major fraction of five glucose units (g.u.) did not co-elute with any of the S2 glycans.

As the triethylamine buffer used for Palpak NP-HPLC is problematic in terms of later MALDI-TOF MS, RP-HPLC was also performed, using also isomalto-oligosaccharides and S2 N-glycans as calibrants, in order to separate fractions prior to mass spectrometry. Ten fractions (**I**–**X**; Fig. 1(B)) containing obvious glycans were isolated and the subsequently acquired spectra (Supporting Information Fig. 1) indicated that Hex_5–10_HexNAc_2_ were present in a number of fractions with retention times compatible with those of the S2 glycans. In addition, a number of putatively methylated species were detected. Other than for the unmodified oligomannosidic glycans, MS/MS was performed in order to verify the putative compositions (Table 1).

**Table 1:** Summary of MALDI-TOF MS analysis of RP-HPLC fractions

*m*/*z* [M + H^+^]	*m*/*z* [M + Na^+^]	Composition	Fraction no.	Notes
1017	1039	H_3_N_2_Me_2_	Fr. IX	MS/MS 176
1313	1335	H_5_N_2_	Fr. IV	Co-elution
1325	1347	H_4_N_2_F_1_Me_2_	Fr. X	MS/MS 607
1341	1363	H_5_N_2_Me_2_	Fr. VII	Prediction
1355	1377	H_5_N_2_Me_3_	Fr. V/VIII	MS/MS 176
1475	1497	H_6_N_2_	Fr. III	Co-elution
1500	1522	H_4_N_3_F_1_	Fr. IX	MS/MS 607
1501	1523	H_5_N_2_F_1_Me_3_	Fr. X	MS/MS 783
1503	1525	H_6_N_2_Me_2_	Fr. VI	MS/MS 176
1517	1539	H_6_N_2_Me_3_	Fr. V/VIII	MS/MS 176
1531	1553	H_6_N_2_Me_4_	Fr. IX	MS/MS 176
1637	1659	H_7_N_2_	Fr. III	Co-elution
1679	1701	H_7_N_2_Me_3_	Fr. VI/IX	MS/MS 176
1799	1821	H_8_N_2_	Fr. I	Co-elution
1827	1849	H_8_N_2_Me_2_	Fr. V	MS/MS 176
1841	1863	H_8_N_2_Me_3_	Fr. VI	MS/MS 176
1961	1983	H_9_N_2_	Fr. II	Co-elution
1989	2011	H_9_N_2_Me_2_	Fr. VI	MS/MS 176
2003	2025	H_9_N_2_Me_3_	Fr. VII	MS/MS 176
2123	2145	H_10_N_2_	Fr. III	Prediction

F, fucose; H, hexose; N, HexNAc.

The *m*/*z* values and predicted compositions of pyridylaminated *Dugesia* N-glycans observed in the collected fractions **I**–**X**. As appropriate, further data aiding identification of the glycan are noted: either MS/MS with an indication of a key diagnostic fragment (see Supporting Information Data for all MS and MS/MS spectra) or co-elution with an oligomannosidic glycan from S2 cells. The *m*/*z* [M + Na^+^] is indicated to facilitate comparison with data from the literature.

### MS/MS analysis of methylated oligomannosidic glycans of *Dugesia*

**Figure 2:**
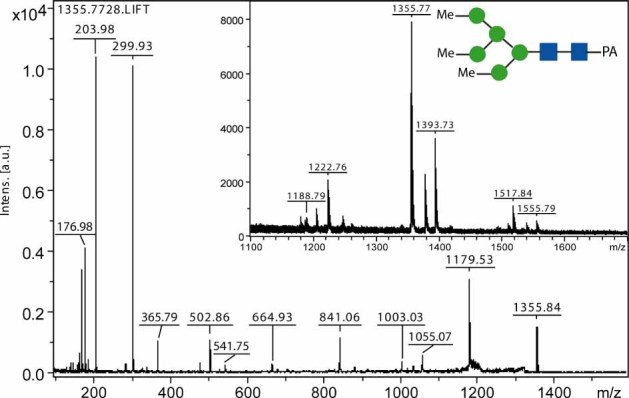
Mass spectrometric analysis of the dominant *Dugesia* N-glycan. The major RP-HPLC fraction (**VIII**) was analysed by MALDI-TOF MS and the dominant species with *m*/z 1355 (see inset) and putative composition Hex_5_HexNAc_2_Me_3_-PA was further examined by MS/MS. The key diagnostic fragments are those of *m*/*z* 176 (methylhexose), 203 (internal HexNAc), 299 (HexNAc_1_-PA), 365 (Hex_1_HexNAc_1_; putatively Manβ1,4GlcNAc), 502 (HexNAc_2_-PA), 541 (Hex_2_HexNAc_1_Me_1_; putatively MeManα1,3Manβ1,4GlcNAc), 664 (Hex_1_HexNAc_2_-PA), 841 (Hex_2_HexNAc_2_Me_1_-PA), 1003 (Hex_3_HexNAc_2_Me_1_-PA; loss of two terminal methylhexose residue), 1055 (Hex_5_HexNAc_1_Me_3_; loss of HexNAc-PA) and 1179 (Hex_4_HexNAc_2_Me_2_-PA; loss of one terminal methylhexose residues). The proposed structure is shown according to the nomenclature of the Consortium for Functional Glycomics (Man, circles; GlcNAc, squares). Of the remaining annotated molecular ions in this fraction, those with *m*/*z* 1188 and 1222 have MS/MS spectra indicating that they are not glycans (present in fractions **VI**–**IX**; see also Supporting Information Fig. 1), whereas the ion with *m*/*z* 1517 corresponds to Hex_6_HexNAc_2_Me_3_.

The major fraction (**VIII**) was found to contain predominantly a glycan with an *m*/*z* value of 1355; under consideration of the biosynthetic pathway for N-glycans in eukaryotes, the MS/MS data suggest that this corresponds to a Man_5_GlcNAc_2_ glycan with methylation of the three terminal mannose residues as indicated by three losses of *m*/*z* 176 and two of *m*/*z* 162 from the parent ion ( Fig. 2). The significance of the presence of a glycan with the same composition and similar fragmentation in fraction **V** is unclear; epimerisation of the core GlcNAc during derivatisation may be an explanation, as an earlier retention time for ManNAc-PA as opposed to GlcNAc-PA has been previously reported.[12]

A number of other glycans are also predicted by interpretation of MS/MS spectra (Supporting Information Fig. 2) to have terminal methylation of mannose residues: trimethylation of putative Man_6–9_GlcNAc_2_ is apparent as is dimethylation of putative Man_3, 6, 8, 9_GlcNAc_2_. For instance, the major components of fractions **VI** (Hex_8_HexNAc_2_Me_3_; *m*/*z* 1841) and **IX** (Hex_3_HexNAc_2_Me_2_; *m*/*z* 1017) are also, on the basis of MS/MS data predicted to contain, respectively, three and two terminal methylhexose residues each. Another glycan in fraction **IX** has the predicted composition Hex_6_HexNAc_2_Me_4_; the fragment of this tetramethylated glycan with *m*/*z* 1017 would be compatible with the presence of two methylated mannose residues linked in series to the mannosylchitobiosyl core.

### Analysis of a late-eluting fraction of *Dugesia* N-glycans

**Figure 3:**
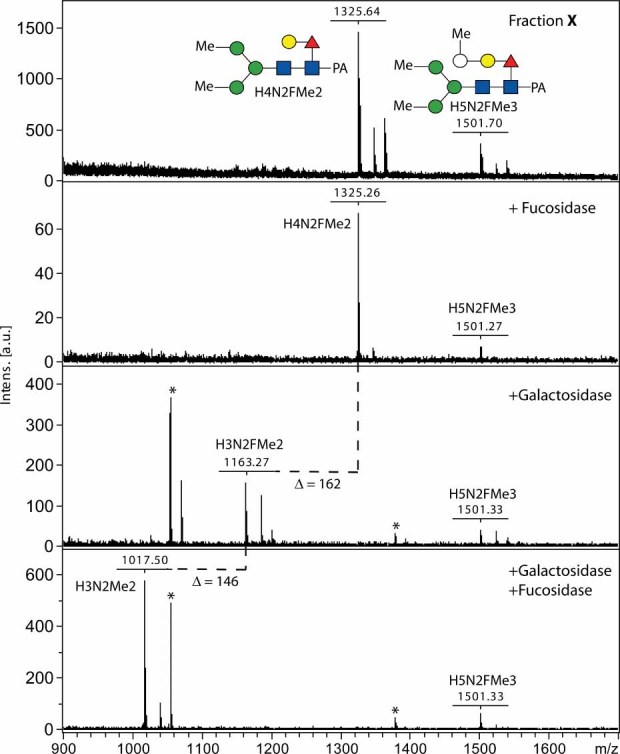
Exoglycosidase digestion of a *Dugesia* galacto-fucosylated N-glycan. The RP-HPLC purified fraction **X** was incubated with either no enzyme, α1,6-fucosidase, β1,4-galactosidase or a combination of fucosidase and galactosidase; the products were analysed by MALDI-TOF MS. Treatment with fucosidase alone resulted in no digestion; however, in the presence of galactosidase, the *m*/*z* 1325 species (Hex_4_HexNAc_2_Fuc_1_Me_2_-PA) was converted to a species with *m*/*z* 1163, consistent with the removal of one hexose. A combination of both galactosidase and fucosidase resulted in a product with *m*/*z* 1017, consistent with the loss of both a galactose and a fucose residue; the *m*/*z* 1501 species (Hex_5_HexNAc_2_Fuc_1_Me_3_-PA) was resistant to this treatment. The *m*/*z* 1054 and 1379 species, marked with an asterisk, are components present in the galactosidase preparation. Proposed structures for both the *m*/*z* 1325 and 1501 glycans, based also on MS/MS data shown in Fig. 4, are depicted according to the nomenclature of the Consortium for Functional Glycomics (Gal, yellow circles; undefined Hex, white circle; Man, green circles; Fuc, red triangle; GlcNAc, blue squares).

Among the fractions putatively containing methylated glycans was fraction **X**; two species with *m*/*z* 1325 and 1501 were detected which could correspond to Hex_4_HexNAc_2_Fuc_1_Me_2_ and Hex_5_HexNAc_2_Fuc_1_Me_3_, respectively ( Fig. 3). In order to examine these glycans further, fraction **X** was subjected to exoglycosidase digestions with either bovine α-fucosidase, which cleaves core α1,6-fucose linkages 20 times more rapidly than core α1, 3-fucose,[13] or a fungal galactosidase, previously shown to be β1,4-specific.[14] Fucosidase alone did not alter the MALDI-TOF MS spectrum; however, in the case of the galactosidase, loss of the *m*/*z* 1325 glycan was accompanied by the appearance of a glycan of *m*/*z* 1163. A provisional conclusion was that a β1,4-galactose residue ‘blocks’ the action of the fucosidase towards Hex_4_HexNAc_2_Fuc_1_Me_2_, indicative of the presence of a Galβ1,4Fuc moiety on the reducing-terminal GlcNAc; this supposition was confirmed by using a combination of both enzymes, which resulted in digestion to a species with *m*/*z* 1017. The putative Hex_5_HexNAc_2_Fuc_1_Me_3_ glycan was not affected by either treatment; considering also the MS/MS data (see below), it is concluded that, on this glycan, the Galβ1,4Fuc modification is ‘capped’ with a methylhexose residue; a precedent for such a modification is the hexose capping of ‘GalFuc’ observed on glycans from keyhole limpet hemocyanin[15] and nematodes.[16] The RP-HPLC elution time of the original fraction (*ca* 30 min) is also compatible with the presence of the ‘GalFuc’ modification, as glycans from *Caenorhabditis elegans* carrying this moiety display a late retention time.[17]

**Figure 4:**
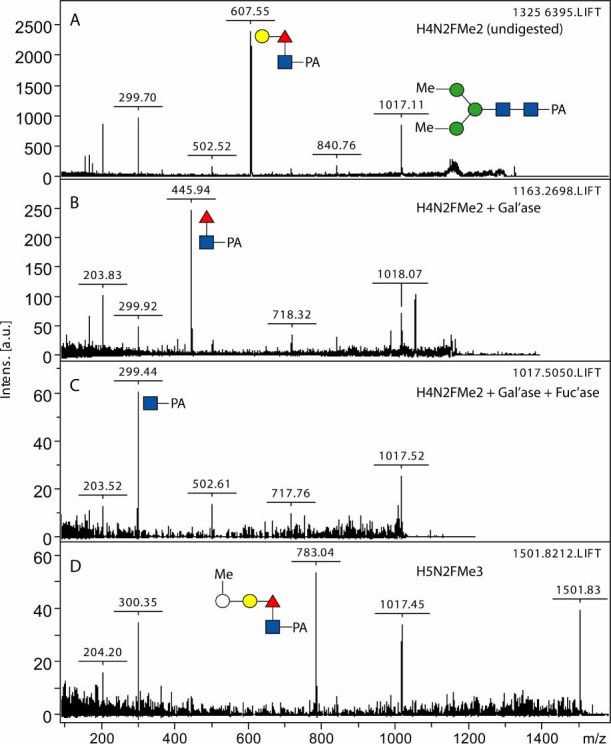
Mass spectrometric analysis of *Dugesia* galacto-fucosylated N-glycans. The late-eluting RP-HPLC fraction (**X**) was analysed by MALDI-TOF MS either without (A and D) or with digestion with galactosidase (B) or a combination of fucosidase and galactosidase (C) ( Fig. 3) and the major species were subject to MS/MS. Diagnostic fragments include those of *m*/*z* 203 (internal HexNAc), 299 (HexNAc_1_PA), 445 (Fuc_1_HexNAc_1_PA), 502 (HexNAc_2_-PA), 607 (Hex_1_Fuc_1_HexNAc_1_PA), 718 (Me_2_Hex_3_HexNAc_1_), 783 (Me_1_Hex_2_Fuc_1_HexNAc_1_PA), 840 (Me_1_Hex_2_HexNAc_2_PA, loss of the ‘GalFuc’ modification and loss of one terminal methylhexose) and 1017 (Me_2_Hex_3_HexNAc_2_PA, loss of ‘GalFuc’).

The undigested and digested forms of fraction **X** were examined by MS/MS. For the two major species in the undigested sample, major fragmentation products of *m*/*z* 607 (Hex_1_HexNAc_1_Fuc_1_PA) and 783 (Hex_2_HexNAc_1_Fuc_1_Me_1_PA) were observed, the former being reminiscent of a fragment from nematode glycans carrying galactosylated core fucose residues.[18] Upon galactosidase and combined galactosidase/fucosidase digestion, the *m*/*z* 607 fragment was no longer observed; indeed, MS/MS of the product of galactosidase digestion resulted in a fragment of *m*/*z* 445 (Fuc_1_HexNAc_1_PA), which was no longer apparent in the product of combined galactosidase/fucosidase treatment ( Fig. 4). It is also concluded, by analogy to other structures in this species and from the fragmentation pattern, that the two putative terminal mannose residues are also methylated in these two glycans. The *m*/*z* 607 fragment was also observed when analysing a glycan in fraction **IX** whose composition is probably Hex_4_HexNAc_3_Fuc_1_ (*m*/*z* 1500; see Supporting Information Fig. 2); however, the amount of this glycan was too low for further analysis.

## DISCUSSION

As shown by MALDI-TOF MS analyses of RP-HPLC fractions, *D. japonica* expresses an unusual range of glycans including standard oligomannosidic, methylated oligomannosidic and at least three galacto-fucosylated N-glycans. As this manuscript was in preparation, a study verifying the presence of the former two glycan categories was published; the authors also showed the presence of a glycan with the same mass as one of the galacto-fucosylated glycans described here, but neither exoglycosidase digestions nor MS/MS analyses were apparently performed and the presence of a ‘GalFuc’ epitope was not postulated.[7] On the other hand, they suggested the presence of a pentose and a fucose or two fucose residues on one of the glycans; however, masses compatible with such modifications, which are conceivable for invertebrates, were not revealed in our analysis. Certainly, both their report and ours agree that the major glycan is a trimethylated species, most probably with the composition Man_5_GlcNAc_2_Me_3_, whereby monosaccharide analyses would indicate 3-*O*-methylation of mannose, a modification conferring resistance to α-mannosidase digestion[7]; our MS/MS data are compatible with a terminal location of the methyl residues. Methylation of terminal mannose residues is known, for instance, on some mollusc N-glycans.[10,19,20]

Our data indicating modification of planaria glycans by Galβ1,4Fuc indicate that this epitope is more widely present than previously thought; until now the only reports of this moiety were on glycans from molluscs, such as squid,[21] octopus[22] and keyhole limpet,[15] as well as from the nematode *C. elegans*.[16] Recently, Galβ1,4Fuc was demonstrated to be the target of a fungal nematoxic lectin, CGL2, and mutant nematodes with defects in either the core α1,6-fucosyltransferase FUT-8 or a novel β1,4-galactosyltransferase GALT-1 were resistant to this lectin.[18] Therefore, it will be interesting to observe whether platyhelminths other than planaria express this epitope, particularly as some trematode and cestode species (e.g. *Schisotoma* spp. and *Echinococcus* spp.) are parasitic and that new therapeutic strategies are being sought.

**Figure 5:**
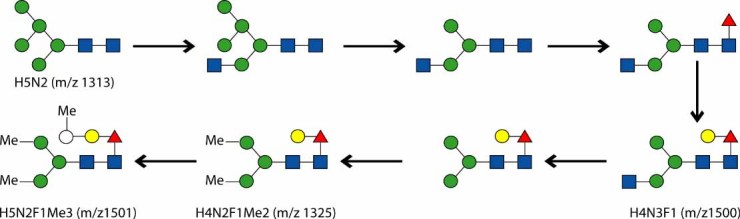
Potential biosynthetic scheme for galacto-fucosylation in *Dugesia japonica.* Glycans are depicted according to the schematic nomenclature of the Consortium for Functional Glycomics. The three glycans predicted by MS/MS to contain the ‘GalFuc’ epitope are indicated with the *m*/*z* of their molecular ions. Although the isomer of Hex_4_HexNAc_3_Fuc_1_ has not been defined (especially whether the non-reducing terminal GlcNAc residue is linked to the α1,3- or α1,6-mannose), we hypothesise that it is a biosynthetic precursor for the other two ‘GalFuc’-modified N-glycans. The proposed biosynthetic scheme is that, after the transfer of the N-glycan and initial trimming by glucosidases and class I mannosidases, *N*-acetylglucosaminyltransferase I acts, thus allowing the Golgi mannosidase II to remove two further mannose residues. The resulting glycan is core fucosylated; the fucose is then capped with galactose and the non-reducing terminal GlcNAc is removed by a Golgi hexosaminidase. After the action of the hexosaminidase, the non-reducing mannose residues are methylated; at some point the ‘GalFuc’ moiety is further capped by a methylhexose residue.

Another aspect of our data is that we can predict some aspects of the glycogenomic capabilities of planaria. For instance, unlike some parasitic protozoa,[23] we expect that planaria contain the full complement of mannosyltransferases required for synthesis of the dolichol-linked precursor for *N*-glycosylation as, at least, a Hex_10_HexNAc_2_ glycan, putatively Glc_1_Man_9_GlcNAc_2_ was detected; also, there should be a core α1,6-fucosyltransferase and a fucoside-modifying β1,4-galactosyltransferase as well as sugar methyltransferases (see Fig. 5 for a potential biosynthetic scheme). Depending on the processing of glycans in the planarial Golgi apparatus, mannosidases and an *N*-acetylglucosaminyltransferase I should be encoded by the genome. However, considering the almost complete absence of non-reducing terminal GlcNAc from the observed glycans, we would postulate that a Golgi hexosaminidase is present, as is the case in insects and nematodes.[17] Preliminary homology-based searching of expressed sequence tags and draft partial genome sequences available from *S. mediterranea* would indeed suggest that homologues of *N*-acetylglucosaminyltransferase I, core α1,6-fucosyltransferase and a fucoside-modifying β1,4-galactosyltransferase are present in at least one related planaria species (data not shown). The detection of a glycan with the composition Hex_4_HexNAc_3_Fuc_1_ would indeed be compatible with the action of *N*-acetylglucosaminyltransferase I being a pre-requisite for core fucosylation and subsequent capping with galactose, as is the case with recombinant *C. elegans* FUT-8 and GALT-1.[24]

Considering that the Galβ1,4Fuc epitope is a target of some galectins, it is relevant to consider the biological roles that these proteins have in other organisms. In mammals, other types of terminal galactose residues are recognised by galectins, which have a number of roles, e.g. in immunity,[25] infection[26] and cellular regulation,[27] whereas in *C. elegans*, LEC-6 is an endogenous ‘GalFuc’ receptor.[28] Therefore, one question is whether galectins of planaria also have such roles and whether, in the absence of obvious endogenous *N*-acetyllactosamine modifications on N-glycans, they are capable of interacting with the Galβ1,4Fuc epitope. It is naturally quite another question to determine whether this modification or any other, such as methylation, has a relevance to the regenerative ability of these organisms, but identification of their N-glycans is a first step in order to design strategies to do so.
