# Supplementary material for: Presence of galactosylated core fucose on N-glycans in the planaria Dugesia japonica
Source: J Mass Spectrom. 2011 Jun;46(6):561–7. doi: 10.1002/jms.1925 (PMC3155867; doi:10.1002/jms.1925)

Presence of galactosylated core fucose on N-glycans in the planaria *Dugesia japonica*

Katharina Paschinger, Ebrahim-Razzazi-Fazeli, Kiyoshi Furukawa and Iain B. H. Wilson

Supplementary Data

Supplementary Figure 1: MALDI-TOF MS of all ten RP-HPLC fractions of *Dugesia japonica* N-glycans. Protonated molecular ions are annotated. The species marked with a asterisk are non-glycan components found in fractions **VI-IX**.

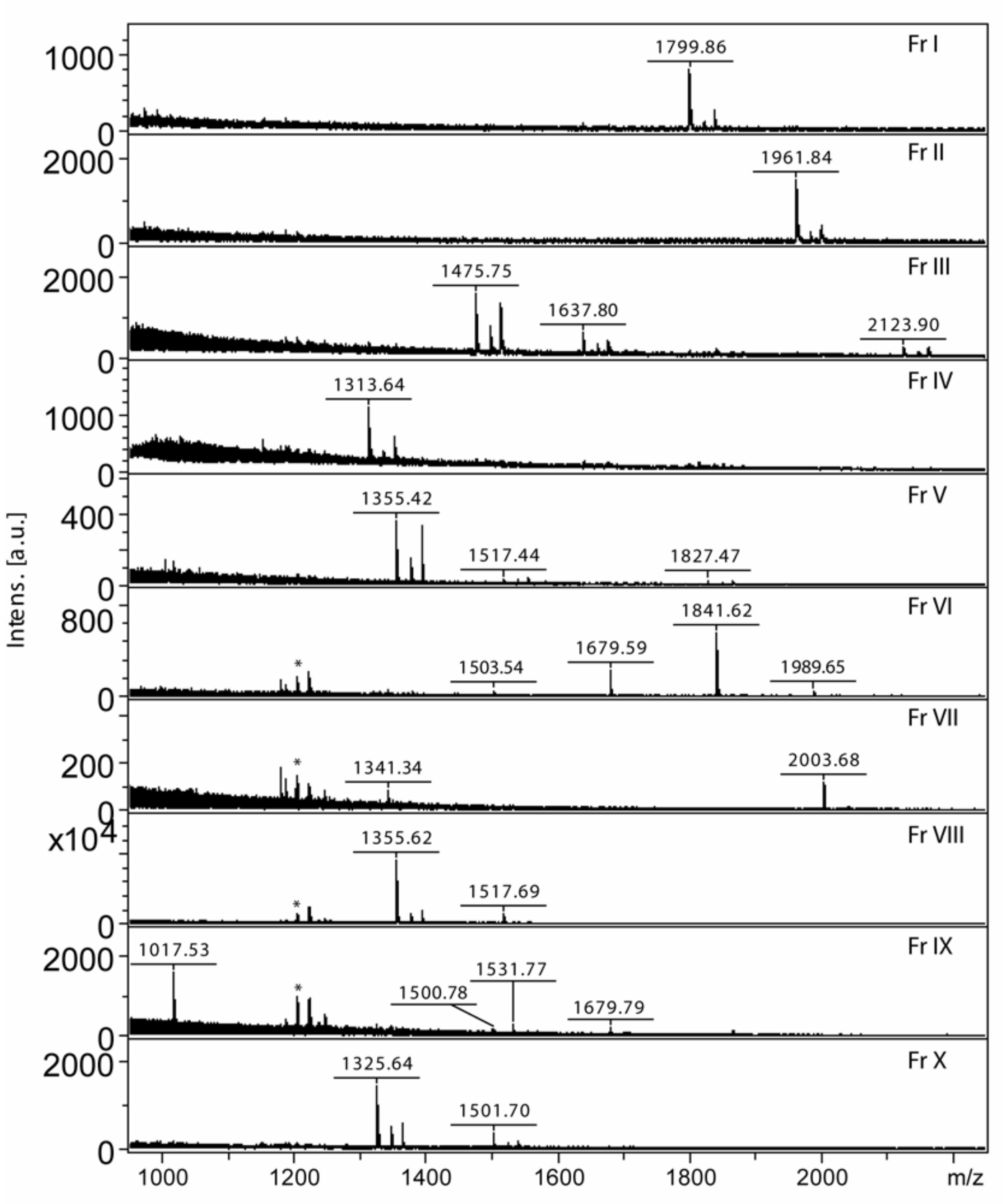

Supplementary Figure 2: MALDI-TOF MS/MS of methylated and fucosylated *Dugesia japonica* N-glycans. Selected PA-labelled fragments of glycans present in fractions **V-IX** are annotated; a fragment of 299 corresponds to the reducing-terminal GlcNAc-PA residue. The fragmentation patterns for glycans in fraction **X** are presented in Figure 4. Putative structures for selected glycans are shown.

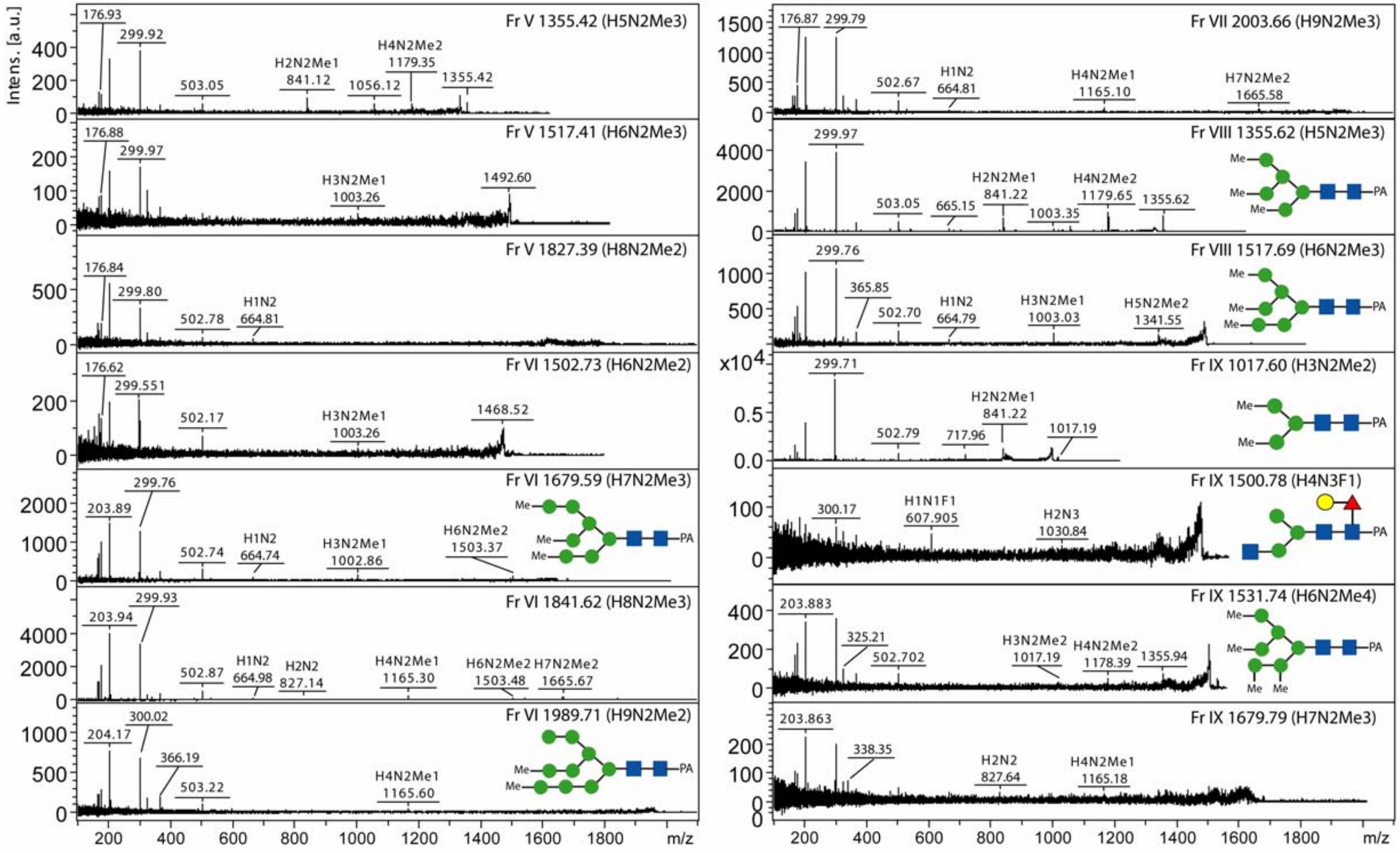

Supplement: Supplementary file 1 [file jms0046-0561-SD1.pdf]
